# Programmed cell death and its role in inflammation

**DOI:** 10.1186/s40779-015-0039-0

**Published:** 2015-05-19

**Authors:** Yong Yang, Gening Jiang, Peng Zhang, Jie Fan

**Affiliations:** Department of Surgery, University of Pittsburgh School of Medicine, Pittsburgh, PA 15213 USA; Department of Thoracic Surgery, Shanghai Pulmonary Hospital, Tongji University School of Medicine, Shanghai, 200433 China; Research and Development, Veterans Affairs Pittsburgh Healthcare System, Pittsburgh, PA 15240 USA

**Keywords:** Inflammation, Necroptosis, Apoptosis, Pyroptosis, Pyronecrosis, NETosis, Autophagy

## Abstract

Cell death plays an important role in the regulation of inflammation and may be the result of inflammation. The maintenance of tissue homeostasis necessitates both the recognition and removal of invading microbial pathogens as well as the clearance of dying cells. In the past few decades, emerging knowledge on cell death and inflammation has enriched our molecular understanding of the signaling pathways that mediate various programs of cell death and multiple types of inflammatory responses. This review provides an overview of the major types of cell death related to inflammation. Modification of cell death pathways is likely to be a logical therapeutic target for inflammatory diseases.

## Introduction

One of the most important factors in the development and homeostasis of organisms is the balance between cell survival and cell death. Early in 1960, apoptosis was considered the only standard programmed cell death form [1, 2], whereas necrosis was mostly considered an ‘accidental’ cell death that occurred in response to physical and chemical insults. Following the progression in cell death research, a tight link was demonstrated between molecularly defined cell death and inflammation. In host defense, programmed cell death can act in a protective manner; the death of infected cells may reduce microbial infections, separate uninfected neighboring cells, and alert the host through danger signals and inflammatory mediators. This review depicts intimate interconnections between cell death and inflammation and the pivotal protein in each special mechanistic module that executes the process of cell death and inflammation.

## Review

### Necrosis, necroptosis, and inflammation

Traditionally, necrosis is considered the primary form of cell death caused by inflammation. Necrosis was historically viewed as an accidental subroutine, largely resulting from very harsh physicochemical stimuli, including abrupt changes in temperature, osmotic pressure, or pH. Necrosis is morphologically identified by the swelling of organelles, increased cell volume, disruption of the plasma membrane, and loss of intracellular content. Necrosis is recognized as a cause of inflammation; the release of intracellular materials, which are termed as damage-associated molecular patterns (DAMPs), can trigger inflammatory reactions. DAMPs are the key to the pathogenesis of sterile inflammation, including gout, atherosclerosis, ischemia-reperfusion, and Alzheimer’s disease. For example, the DAMP molecule high-mobility group box 1 (HMGB1) can be released from necrotic cells and, in turn, stimulates neighboring cells via the receptor for advanced-glycation end-products (RAGE) to express proinflammatory cytokines, chemokines, and adhesion molecules, therefore inducing inflammation [3]. Recent studies using genetic approaches [4–7] and chemical inhibitors of necrosis [4, 8, 9] demonstrated the existence of multiple pathways of regulated necrosis.

**Fig. 1:**
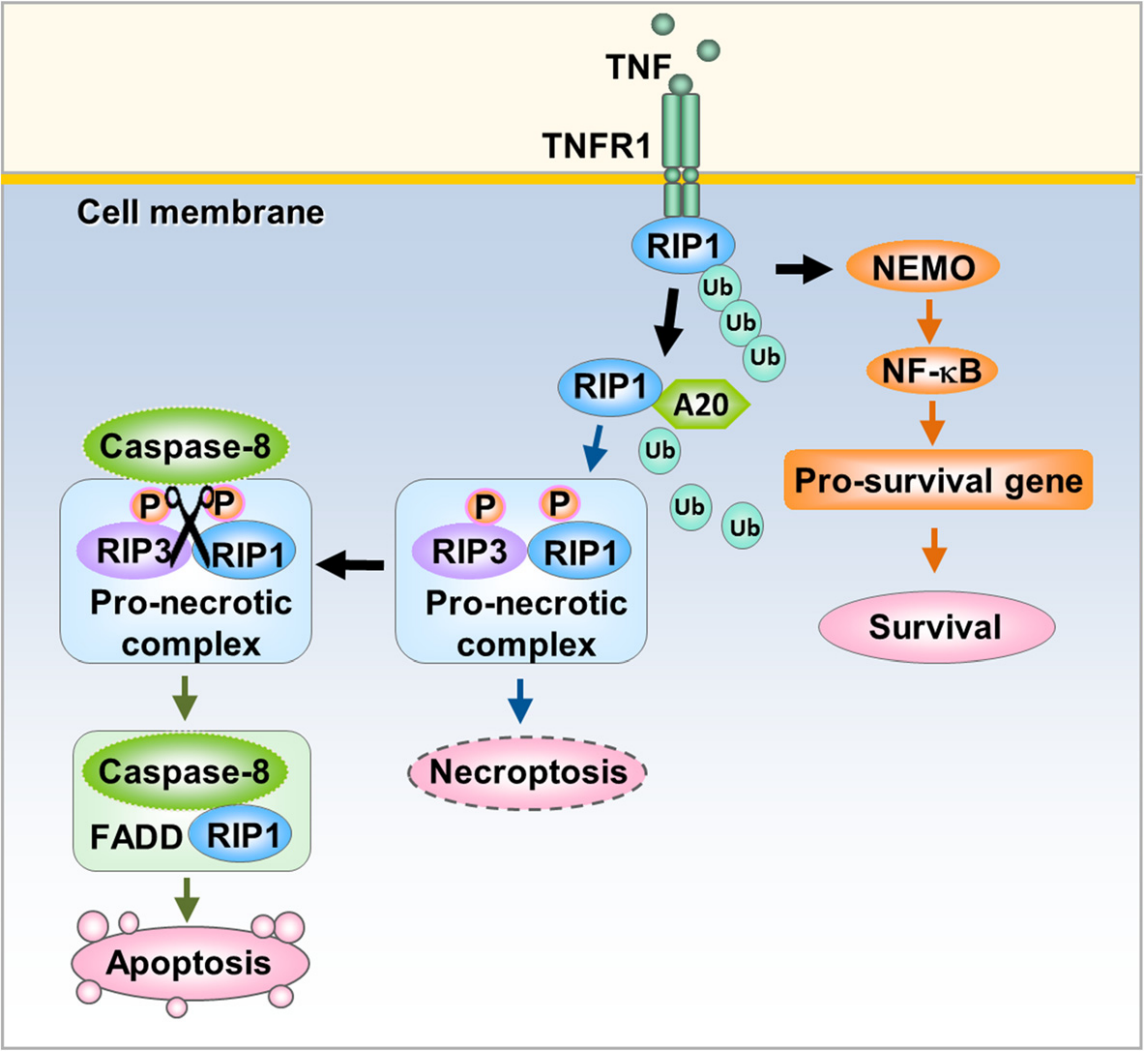
Model of TNF receptor signaling regulation of cell fate. Upon the binding of TNF to its receptor TNFR1, RIP1 is recruited to TNFR1 and is subsequently ubiquitinated. The polyubiquitinated RIP1, in turn, binds to NEMO, the regulatory subunit of NF-kB, to promote NF-κB activation, which leads to the induction of pro-survival genes to counter the death signals. Cell survival is a result of this pathway. The polyubiquitinated RIP1 can also migrate to the cytoplasm, where RIP1 is de-ubiquitinated by A20, the de-ubiquitylating enzyme. RIP1 and RIP3 can then form a pro-necrotic complex followed by phosphorylation on both kinases and induction of necroptosis. In circumstances in which caspase-8 is activated, RIP1 and RIP3 can be cleaved by caspase-8, and the pro-necrotic complex is blunted, which stimulates the cell to undergo apoptosis

Among the pathways of regulated necrosis, necroptosis is currently most frequently mentioned and investigated. Generally, necroptosis is defined as cell death mediated through a pathway that depends on the receptor-interacting protein kinase (RIP)1-RIP3 complex and that can be inhibited by Necrostatin-1 (Nec-1) [10] (Fig. 1). Necroptosis is induced by a class of death receptors that includes tumor necrosis factor receptor (TNFR)1, TNFR2, and Fas. Of these, the TNF-α/TNFR-induced pathway is the most widely studied. Binding of TNF-α to the extracellular portion of TNFR1 causes allosteric changes in the intracellular portion of TNFR1 followed by the release of the silencer of death domains (SODD) from the intracellular domain of TNFR1 [11]. TNFR1 and TNFR2 form complex I containing a death domain (e.g., TNF-α receptor-associated death domain (TRADD)), RIP1, Fas-associated death domain (FADD), and several E3 ubiquitin ligases, such as TNF-α receptor associated factor 2/5 (TRAF2/5) and inhibitor of apoptosis proteins (IAPs) cIAP1 and cIAP2 [12]. RIP1 is initially recruited to complex I and is polyubiquitinated by TRAF2/5, cIAP1, and cIAP2 [13, 14]. Because RIP1 exhibits a biphasic effect based on its ubiquitination state, complex I is situated at the crossroads of cell survival and death. Deubiquitination of RIP1 can inhibit the NF-κB pathway, which promotes cell death pathways. Whether TRADD is required for necroptosis potentially depends upon the type of stimulus. TNFR1 activation together with the absence of c-IAPs (IAP antagonist treatment), translation inhibition (cyclohexamide treatment), or RIP1 deubiquitination by the deubiquitinating enzyme (DUB) CYLD may promote the translocation of RIP1 to a secondary cytoplasmatic complex, Complex II [15–17]. Complex II is formed by the death domain containing protein FADD, caspase-8 and cFLIP. Complex II may activate either apoptotic or necroptotic downstream signaling pathways. Activation of caspase-8 drives complex II into a pro-apoptosis state by cleaving RIP1 and RIP3. However, when the apoptosis pathway is inhibited, a complex named the “necrosome” is formed (Fig. 1). The necrosome is primarily composed of RIP1 and RIP3 and distinctly enhances necroptosis [18].

The pseudokinase MLKL is a substrate of RIP3 required for necroptosis [4, 19]. Unlike its previous discovered function in regulating mitochondrial fission, MLKL recruitment and phosphorylation caused by RHIM-dependent oligomerization and intramolecular RIP3 autophosphorylation [20, 21] results in an activated state able to induce necroptosis [22]. Furthermore, several studies have deciphered a role for MLKL in necroptosis. MLKL oligomerization induced by RIP3 and plasma membrane localization is associated with its cytotoxicity [23–26]. MLKL binds to phosphatidylinositol phosphates (PIPs) [23, 25] and subsequently modifies sodium or calcium influx through ion channels, thereby increasing osmotic pressure and promoting plasma membrane rupture [24, 26, 27].

The mechanism by which the necrosome causes cell death remains unclear. Necroptosis shares some identical sub-cellular events with necrosis, such as oxidative burst, mitochondrial membrane hyperpolarization, lysosomal membrane permeabilization, and plasma membrane permeabilization. However, the mechanisms underlying those processes might be different [28]. Reactive oxygen species (ROS) potentially lead to cell death by directly oxidizing or triggering various downstream pathways in the mitochondria [29–31]. RIP3 accelerates mitochondrial ROS production and mitochondrial metabolism through the activation of a series of metabolism-related enzymes, including NADPH and JNK [32, 33]. Mitochondria also contribute to necrotic cell death through an ADP/ATP-related pathway in addition to ROS production. Adenine nucleotide translocase (ANT), an ADP/ATP carrier located in the inner mitochondrial membrane, is required for the synthesis of ATP in the mitochondria. RIP1-dependent inhibition of ANT is reportedly involved in the programmed necrosis induced by TNF-α and zVAD-fmk, whereas the later potentially blocks the ability of ANT to transport cytoplasmic ADP and thereby induces massive ATP depletion in mitochondria. The activity of ANT is potentially affected by interactions with VDAC and cyclophilin D (CYPD). Two other potential executional proteins are cPLA2 and lipoxygenase (LOXs). cPLA2 plays an important role in TNF-α-induced necrotic cell death in L929 cells and MEFs [34]. LOXs acts as a downstream effector of PLA2 and leads to the disruption of organelle and plasma membranes [35]. LOXs is reportedly involved in both apoptosis and necrosis induced by TNF-α, although the exact mechanism has yet to be defined [36, 37].

Necroptosis is able to trigger inflammation. This effect has been observed in a study using mice with deletion of FADD [38] or Casp8 [39] in intestinal epithelial cells (IECs) in which RIP3-dependent cell death caused intestinal inflammation. RIP3-mediated necroptosis may play a role in the pathogenesis of Crohn’s disease, as evidenced by the high RIP3 expression in Paneth cells of these patients [39]. Necroptosis has been found to stimulate the immune system to elicit inflammatory responses and has also been characterized in animal models of acute pancreatitis, ischemic injury, and neurodegeneration [9, 40–42]. RIP3^−/−^ mice are protected from systemic inflammation caused by TNF stimulation and experimental sepsis induced by cecal ligation and puncture (CLP) [43, 44]. RIP1 and RIP3 also play crucial roles in the pathogenesis of *Salmonella enterica serovar* and *S. typhimurium* infection [45]. Necrotic macrophages have been observed in atherosclerosis lesions from both human patients and animals [46]. RIP3-dependent necroptosis is a key driver for inflammation in atherosclerosis; RIP3 deficiency alleviates macrophage necrosis in advanced atherosclerosis lesions in atherosclerosis-prone LDL-R^−/−^ or ApoE^−/−^ mice [47]. The contribution of RIP1-dependent necroptosis to multiple organ failure has also been observed in models of ischemia reperfusion (IR) and can be rescued by Nec-1 inhibitor [48–50]. In addition, necroptosis has been shown to contribute to neuronal damage in neonatal brain injury [51].

Taken together, necrosis and necroptosis are endogenous triggers of inflammation that influence host disease outcomes. Determining the relative contribution of necroptosis-dependent and -independent pathways in inflammation may lead to new and more specific therapeutic targets.

### Apoptosis and inflammation

Apoptosis is one of the major types of cell death and has been well defined for many years. Two independent apoptotic signaling cascades, the extrinsic and intrinsic pathways, have been distinguished [52]. The extrinsic pathway is triggered by binding of Fas plasma membrane death receptor to Fas ligand (Fas-L) and other similar receptors, such as TNFR 1 and its relatives [53]. Fas-L combines with Fas to form a death complex. The Fas/Fas-L composite recruits death domain-containing protein (FADD) and pro-caspase-8, aggregating to become the death-inducing signaling complex (DISC). Consequently, the protein complex activates pro-caspase-8, which proceeds to trigger pro-caspase-3, the penultimate enzyme for the execution of the apoptotic process [54]. The intrinsic pathway also leads to apoptosis but under the control of mitochondrial pro-enzymes. When a cell is stimulated by either extracellular stimuli or intracellular signals, the outer mitochondrial membranes become permeable to internal cytochrome c, which is then released into the cytosol. Cytochrome c associates with the adaptor protein Apaf-1 to form the apoptosome, which triggers downstream caspase 9 [55]. Once activated, caspases-8, −9, and −10 process the executioner caspases-3 and −7. Mature caspases-3 and −7 cleave a large set of substrates, ultimately resulting in the characteristic morphological and biochemical hallmarks of apoptosis, such as phosphatidylserine exposure, nuclear condensation, membrane blebbing, and genomic DNA fragmentation.

Many factors and signaling pathways that are activated by inflammation are involved in the regulation of cell apoptosis. Absent in melanoma 2 (AIM2), a member of the pattern recognition receptors (PRRs) in the cytoplasm, has been found to activate caspase-3 in parallel with caspase-1 [56]. AIM2 can recognize DNA released by the cytosolic bacteria [57], whereas NLRP3, another member of the cytoplasmic PRRs, responds to the bacterial pore-forming toxin nigericin [58], both of which elicit apoptotic caspase activation [59, 60]. Apoptotic responses can be observed in wild type cells responding to AIM2 or NLRP3 stimuli [58]. AIM2 and NLRP3 inflammasome-dependent apoptosis requires caspase-8, which is recruited to the inflammasome through interaction between its DED domains and the PYD of apoptosis-associated speck-like protein containing a caspase activation and recruitment domains (CARD), an adaptor molecule of the inflammasome [57, 58, 61]. In contrast, BCL-2 can negatively regulate NLRP3 inflammasome activation by preventing the cytosolic release of mitochondrial DNA [62].

Apoptotic cells can expose “eat me” signals, which are either newly expressed molecules or existing molecules modified by oxidation, to initiate phagocytosis of the apoptotic cells [63]. The process of phagocytosis of apoptotic cells represents an anti-inflammatory mechanism. Phosphatidyl serine (PS) localized to the outer leaflet of the plasma membrane is the predominant “eat me” molecule upon apoptosis [63, 64]. Specific molecules such as milk fat globule epidermal growth factor 8 (MFG-E8) links PS to phagocyte a_v_b_3_ integrin [63], whereas growth-arrest-specific 6 (GAS6) links PS to the receptor tyrosine kinase MER [63]. PS acts as a ligand for the T-cell immunoglobulin domain and mucin domain (TIM)-4 molecule on macrophages and dendritic cells (DC) [65], and TIM-4 helps promote the uptake of apoptotic cells [66]. Two other molecules, brain-specific angiogenesis inhibitor 1 (BAI1) and stabilin-2, have also been shown to mediate uptake of apoptotic cells via recognition of PS [67, 68].

Apoptotic cells are rarely detected under physiological conditions, but the presence of uncleared apoptotic cells has been linked to several different diseases, including infection and inflammation. PAMPs and DAMPs are detected by the tissue-resident cells in response to an acute infection or tissue injury. Next, leukocytes aggregate near to the site of inflammation; innate immune cells, such as neutrophils, are often the first cells to appear, whereas mononuclear cells and macrophages accumulate later [69]. This initial robust immune response is designed to destroy invading pathogens and enhance tissue repair [70, 71]. After eliminating the initial threat, leukocyte recruitment ceases, and the previously recruited cells are disposed. The main clearance route of leukocytes is local neutrophil apoptosis and subsequent phagocytosis [72, 73], although they can be cleared through transepithelial migration into the airway lumen in the context of lung inflammation [74] or via lymphatic vessels [75]. The phagocytosis of pathogens, such as *Escherichia coli* or *Staphylococcus aureus*, promotes neutrophil apoptosis following neutrophil recruitment, which is termed phagocytosis-induced cell death (PICD) [76]. This response is believed to be primarily protective for the host, and incidentally, pharmacological acceleration of neutrophil apoptosis is protective in pneumococcal meningitis by reducing the incidence of brain hemorrhage [77]. The failed clearance of apoptotic neutrophils can lead to a prolonged inflammatory response, and this phenomenon has been observed in disease, including chronic obstructive pulmonary disease (COPD) [78], pulmonary fibrosis [79] and cystic fibrosis [80]. The production of ROS by neutrophils involves this impaired phagocytosis process, in which ROS activate the GTPase RHOA in surrounding phagocytes and reduces apoptotic cell engulfment by neighboring cells [81–84]. Alveolar macrophages from patients with severe asthma and children with poorly controlled asthma are defective in clearing apoptotic cells [85, 86]. As the mainstay of treatment in asthma, corticosteroids not only induce eosinophil apoptosis [87] but also enhance monocyte-derived macrophage engulfment [88]. The mechanism underlying the enhanced clearance seems dependent on the binding of protein S to apoptotic cells and the upregulation of tyrosine-protein kinase MER on the surface of macrophages [89]. Recently, airway epithelial cells have been found to be capable of engulfing neighboring apoptotic cells, and deficiency of this engulfing function increases pro-inflammatory mediator production and exacerbates airway inflammation [90]. Apoptotic cells are well established to induce the synthesis of anti-inflammatory mediators such as TGF-β, prostaglandin E2, and platelet activating factor by macrophages [91, 92].

To summarize, contrary to traditional model, specific PRRs may activate apoptotic signaling pathways. More importantly, the clearance of apoptotic cells and neutrophil apoptosis in the host further affects inflammation. Therapeutic induction of neutrophil apoptosis at the inflammatory site may be a powerful pro-resolution intervention and could fulfill the clinical need to prevent the harmful consequences of inflammation.

### Pyroptosis and inflammation

Pyroptosis is a form of cell death that depends on caspase-1 activation. Pyroptosis features rapid plasma-membrane rupture and release of proinflammatory intracellular content. Cell lysis during pyroptosis results from caspase-1-mediated processes [93–101]. Plasma membrane pores dependent on caspase-1 dissipate cellular ionic gradients, producing a net increased osmotic pressure, water influx, cell swelling, and eventual osmotic lysis, followed by release of inflammatory intracellular content [102]. Cell death due to pyroptosis results in a measurable cellular size increase and cleavage of chromosomal DNA [95, 97, 102–105].

The inflammasome, a caspase-1-containing complex that activates the proinflammatory cytokines IL-1β and IL-18 and results in proinflammatory cell death, is one of the drivers of pyroptosis. The inflammasome activates caspase-1 through a Nod-like receptor (NLRP1, 3, 6, 7, 12, NLRC4), AIM2, or Pyrin, all of which contain a CARD or pyrin domain (PYD) [106, 107]. Many inflammasomes recruit the ASC adaptor via homotypic interactions. Additional ASC molecules are incorporated via CARD-CARD and PYD-PYD interactions, until all ASC molecules are collected into a single focus. The recruitment of procaspase-1 into the ASC focus via CARD-CARD interactions results in its dimerization and proximity-induced autoproteolytic processing into the p10 and p20 subunits. This processed and catalytically active caspase-1 cleaves pro-IL-1β and pro-IL-18.

**Fig. 2:**
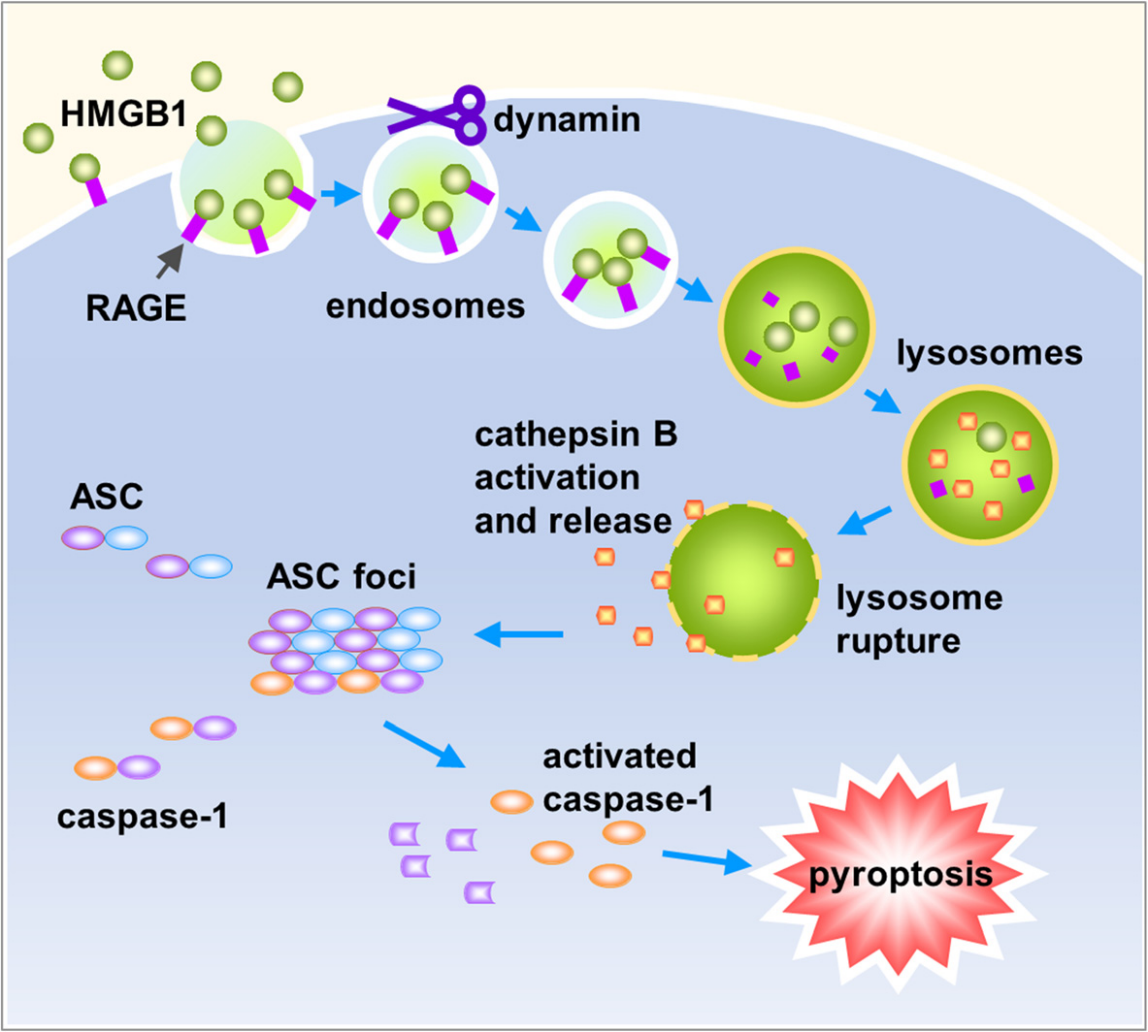
Model of macrophage endocytosis of HMGB1 induces pyroptosis. HMGB1 acting through RAGE on macrophages triggers dynamin-dependent endocytosis of HMGB1, which in turn initiates a cascade of cellular and molecular events. These include CatB activation and release from ruptured lysosomes followed by pyroptosome formation and caspase-1 activation, which promotes HMGB1-induced pyroptosis

We recently reported that HMGB1 acting through RAGE on macrophages (or macrophage membrane) triggers dynamin-dependent endocytosis of HMGB1, which in turn initiates a cascade of cellular and molecular events. These events include cathepsin B activation and release from ruptured lysosomes, followed by pyroptosome formation and caspase-1 activation, which serves as a mechanism underlying the HMGB1-induced macrophage pyroptosis (Fig. 2) [108].

A recent study demonstrated that after pyroptosis, ASC specks accumulate in the extracellular space, where they promote further maturation of IL-1β [109]. In addition, phagocytosis of ASC specks by macrophages induces lysosomal damage and nucleation of soluble ASC as well as activation of IL-1β in recipient cells [109]. These findings indicate that pyroptotic cell-released inflammasomes serve as danger signals promoting enhanced activation of macrophages.

Pyroptotic cells secrete the inflammatory cytokines IL-1β and IL-18 following caspase-1 activation. IL-1β is a potent endogenous pyrogen that stimulates fever, leukocyte tissue migration and expression of diverse cytokines and chemokines [110]. IL-18 induces IFNγ production and is important for the activation of T cells, macrophages and other cell types [111]. Cytokine secretion occurs through caspase-1-dependent pores in the plasma membrane. Pharmacological inhibition of cell lysis does not prevent caspase-1-dependent pore formation and cytokine secretion, suggesting that lysis is not required for the release of active IL-1β and IL-18 [102]. Thus, cytokine secretion and cell lysis are both downstream consequences of caspase-1-dependent pore formation. Notably, caspase-1 activation cannot trigger pyroptosis in all cell types; specifically, epithelial cells use caspase-1 activation to prevent cell death [112]. For example, caspase-1 activation stimulates lipid production and membrane repair in response to the pore-forming toxins aerolysin and α-toxin [112].

In addition to caspase-1, caspase-11 has also been found to be involved in pyroptosis [113–115]. A recent study revealed that caspase-11 participates in the process of non-canonical inflammasome activation downstream of a cytosolic ligand released from bacteria [116, 117].

Pyroptosis may protect against infection and induces pathological inflammation. However, exuberant or inappropriate caspase-1 activation and pyroptosis can be detrimental. During infection, caspase-1 activation helps to clear pathogens, such as *Salmonella* [118, 119], *Francisella* [120], *Legionella* [101, 121], *Shigella* [122], *Anaplasma phagocytophilum* [123], *Burkholderia thailandensis* [124], *Burkholderia pseudomallei* [125] and *Listeria* [126]. Mutations in NLR proteins can lead to improper caspase-1 activation and can cause hereditary autoinflammatory syndromes [127]. Moreover, caspase-1 is involved in the pathogenesis of several diseases characterized by inflammation and cell death, including myocardial infarction [128], cerebral ischaemia [129], neurodegenerative diseases [130], inflammatory bowel disease [131], and endotoxic shock [132].

As one of the most recently recognized types of cell death, pyroptosis exhibits a particular relationship with common pathogens, and clinic inflammatory disease for caspase-1 connects to both cell death and pro-inflammation directly. Pyroptosis and other caspase 1-dependent processes are therefore relevant to our understanding of the pathophysiology of inflammatory disease.

### Pyronecrosis and inflammation

Pyronecrosis is another necrosis-like cell death process that is independent of caspase-1 and caspase-11 but is dependent on ASC and lysosomal protein cathepsin B. Pyroptosis results in the cellular secretion of the pro-inflammatory mediator HMGB1 [133]. Recent studies have demonstrated that pyronecrosis can be induced by several pathogens, including *Neisseria gonorrhoeae* [134], *Toxoplasma gondii parasitophorous* [135], *Bacillus anthracis lethal toxin* [136] and *Staphylococcus aureus* [137]. The mechanism underlying pyronecrosis remains unclear at present and requires further investigation.

### NETosis and inflammation

NETosis is a special form of polymorphonuclear neutrophil (PMN) death that releases neutrophil extracellular traps (NETs) [138]. NETs are web-like structures released by neutrophils that are composed of decondensed chromatin in complex with different neutrophil proteins that can capture, neutralize, and kill microbes. These large extracellular structures provide a physical barrier to prevent microbial dissemination and increase the local concentration of antimicrobial effectors [139, 140]. There are two types of NETosis that can be distinguished by the occurrence time as early and late. The more frequently observed type is late NETosis, as NET release via cell death is a slow process (120–240 min) and is defined as suicidal NETosis. This form of suicide is an NADPH oxidase–dependent cellular death process requiring chromatin decondensation, followed by nuclear envelope disintegration and mixing of nucleic acids and granule proteins within a large intracellular vacuole [141]. However, it remains unclear how oxidants participate in the dismantling of the nuclear envelope and mixing of the NET components. Classically, suicidal NETosis occurs following stimulation by phorbol myristate acetate (PMA) through activation of protein kinase C and the Raf–mitogen-activated protein kinase (MEK)–extracellular signal-regulated kinase (ERK) pathway. NADPH assists in the translocation of neutrophil elastase from cytosolic granules into the nucleus, where it aids in chromatin breakdown via histone cleavage. Myeloperoxidase (MPO) is required for chromatin and nuclear envelope breakdown and granular mixing within the NET vacuole. One hundred twenty minutes after intracellular NET formation, the neutrophil outer membrane ruptures, and the mature NET is extruded.

The early form of NETosis occurs rapidly in response to a pathogen, e.g., after *in vitro Staphylococcus aureus* stimulation for 5–60 min. Early NETosis has also been termed vital NETosis in some studies [142]. In general, NETosis begins when the nucleus loses its characteristic lobulated architecture. Subsequently, nuclear membranes disassemble, and the chromatin decondenses into the cytoplasm while the plasma membrane remains intact. Finally, the plasma membrane bursts, leading to NET released [138]. This process is mainly dependent on ROS, such as superoxide generated by the NADPH oxidase Nox2. This mechanism spares the PMN outer membrane, thereby allowing the PMN to continue to function, even to the point of becoming anuclear. There are three major differences between suicidal NETosis and vital NETosis, including the nature of the inciting stimuli and the timing, the functional capacity of the PMNs during NET release, and the mechanisms employed to make and release NETs. In addition to PMN, NETosis has also been observed in eosinophils and mast cells [143]. Therefore, the more generalized term ‘ETosis’ maybe more accurate [144].

NETs can kill a number of pathogenic bacteria directly, beyond just capturing and immobilization [145–148]. Studies demonstrate that NETs can inactivate bacterial virulence factors, such as IpaB from *S. flexneri* [138]. NETs may also serve to opsonize certain fungi, such as *A. fumigatus* via long pentraxin 3 [149]. NETs generated from PMNs can inhibit the growth of *Aspergillus* [145] and kill *C. albicanscan*, even the opportunistic pathogen *P. aeruginosa* [150]. The gram-negative bacterium *K. pneumoniae* is not sufficient to induce NETosis in isolated neutrophils *ex vivo* but is a good inducer in a mouse lung infection model [151]. Human immunodeficiency virus (HIV)-1 has been shown to induce NETosis through a cell death pathway [152]. Feline leukemia virus (FeLV) was able to inhibit neutrophil activation by inhibiting the activation of PKC to reduce ROS production [153].

Numerous types of inflammation are associated with NETs and NETosis. NETs are observed in acute lung injury (ALI) models of both infection- or sterile- related by influenza virus [154, 155], bacteria or bacterial component LPS [156–158], fungi [148, 159, 160], and transfusion [161, 162]. Among them, human neutrophil antigen (HNA)-3a causes the most severe transfusion-related ALI and has been shown to promote NETosis in human neutrophils *in vitro* [161]. Extracellular neutrophil elastase release via NETosis may be an important cause of lung tissue damage and cystic fibrosis progression [163]. NETs have been shown to form scaffolds in circulation that promote thrombus formation by interacting with the endothelium, platelets, coagulation factors and red blood cells, which cause deep vein thrombosis. IL-8 and ROS release from endothelial cells can recruit and trigger neutrophils to form NETs, which subsequently promote damage to the endothelium through the binding of histones [164].

As a specific cell death type for neutrophils, NETosis help capture numerous pathogenic bacteria and virus. Further insight into the interaction between NETs and invaders would deepen the understanding of the inflammation process. Furthermore, NETotic products could be treated as prognostic biomarkers for inflammatory disorders, and whether the produces correlate with clinical outcome in a variety of diseases requires further translational investigation.

### Autophagy and inflammation

Autophagy is a genetically regulated and evolutionarily conserved pathway for the degradation of subcellular components [165, 166]. Autophagy has previously been classified as a form of programmed cell death to describe a form of caspase-independent necrosis-like cell death associated with the accumulation of autophagosomes in cells [167]. This classification is now controversial, and the casual relationship between autophagy and cell death remains uncertain [168, 169].

Autophagy formation begins when an autophagic isolation membrane (also known as a phagophore) engulfs a portion of cytoplasm [170]. Beclin 1, the serine/threonine protein kinase ULK1, autophagy-related LC3 proteins, and γ-aminobutyric acid receptor-associated proteins are key regulators of phagophore formation [170]. A phagophore sequesters captured cytoplasmic cargo, and a double-membraned autophagosome is formed following elongation and closure. Autophagosome formation is largely controlled by mammalian target of rapamycin (mTOR). Inhibition of mTOR leads to the interaction between ULK1 and AMPK [171, 172], which in turn recruits the type III PI3 kinase VPS34 to promote the development of autophagosome [173, 174]. The degradation of the captured cargo begins when the double-membraned autophagosome matures into a single membrane-delimited autolysosome [175, 176]. Following this step, lysosomes can be recycled from autolysosomes, thereby permitting the cell to reuse a critical component required for further autophagy.

PRR signaling induced by PAMPs and DAMPs can activate autophagy. For instance, TLRs can cooperate with autophagy in response to PAMPs [177, 178], and NLRs interact with ATGs to localize autophagy [179, 180]. Inflammatory cytokines such as IL-1 family members [181, 182] and IFNγ [183–185] are also involved in the activation of autophagy, whereas T_H_2 cell-associated cytokines, IL-4 and IL-13, inhibit autophagy [184].

Multiple studies have confirmed the important role of autophagy during the infection process. Autophagy protects organism from infectious disease by degrading intracellular bacteria, viruses, and protozoan pathogens [186–188].

The role of autophagy in regulating inflammation has been demonstrated in Crohn’s disease and sepsis. Crohn’s disease is a type of chronic inflammation. Polymorphisms in the genes encoding the autophagy-related proteins Atg2a, Atg4a, Atg4d, death-associated protein, immunity-related GTPase family M protein (IRGM), and ULK-1 have been found to be associated with susceptibility to Crohn’s disease [189–191]. NOD2 mutations cause impairment in autophagosome induction and bacterial clearance [179]. Autophagy formation downstream of NOD2 activation controls IL-1β and IL-6 release [192, 193] and results in the tolerogenic presentation of commensal bacterial components on MHC class II complexes in dendrite cells [180]. Inhibition of autophagy in septic mice boosts inflammatory cytokine levels and increases mortality. This effect may due to the failure to clear damaged or dysfunctional mitochondria, which activate the NLRP3 inflammasome [194]. We have recently demonstrated that hemorrhagic shock (HS) acting through HMGB1/TLR4 signaling upregulates NOD2 expression in alveolar macrophages (AM) and subsequently sensitizes AM to the NOD2 ligand MDP, which leads to exacerbated inflammation in the lung. Moreover, upregulated NOD2 signaling induces autophagy in AM, which in turn negatively regulates lung inflammation via a mechanism that involves suppression of NOD2-RIP2 signaling and inflammasome activation. PMNs counteract this anti-inflammatory effect of autophagy via a NAD(P)H oxidase-derived ROS mechanism; therefore, PMNs enhance post-HS lung inflammation [195].

Although the relationship between autophagy and cell death remains uncertain, several members of the inflammation process are involved in autophagy. The function of autophagy in related inflammatory diseases requires further investigation. A better understanding of the relevance of the contribution of autophagy to inflammatory diseases has great clinical potential.

## Conclusion and prospective

Emerging evidence has demonstrated the tight links between cell death and inflammation. A better appreciation of the cross-regulatory relationships between different forms of cell death and pathways will be crucial for understanding their roles in the inflammation process. It is important that we realize the therapeutic possibility of targeting programed cell death in patients. Our understanding of the molecular pathways of programed cell death will allow the development of reagents that control cell death, thereby serving as a novel strategy for interventions in inflammatory diseases. Some types of cell death that do not seem to be related to inflammation may also be considered in future studies in light of their possible interaction with inflammation; these approaches will help us better understand the entire inflammatory process network.

## Abbreviations

AIM: Absent in melanoma
ALI: Acute lung injury
AM: Alveolar macrophages
ANT: Adenine nucleotide translocase
BAI: Brain-specific angiogenesis inhibitor
CARD: Caspase activation and recruitment domains
CLP: Cecal ligation and puncture
COPD: Chronic obstructive pulmonary disease
CYPD: Cyclophilin D
DAMPs: Damage-associated molecular patterns
DC: Dendritic cells
DISC: Death-inducing signaling complex
ERK: Extracellular signal-regulated kinase
FADD: Fas-associated death domain
Fas-L: Fas ligand
FeLV: Feline leukemia virus
GAS: Growth-arrest-specific
HIV: Human immunodeficiency virus
HMGB1: High-mobility group box 1
HNA: Human neutrophil antigen
HS: Hemorrhagic shock
IAPs: Inhibitor of apoptosis proteins
IECs: Intestinal epithelial cells
IR: Ischemia reperfusion
IRGM: Immunity-related GTPase family M protein
LOXs: Lipoxygenase
MEK: Raf-mitogen-activated protein kinase
MFG-E: Milk fat globule epidermal growth factor
MPO: Myeloperoxidase
mTOR: Mammalian target of rapamycin
NETs: Neutrophil extracellular traps
PICD: Phagocytosis-induced cell death
PIPs: Phosphatidylinositol phosphates
PMA: Phorbol myristate acetate
PMN: Polymorphonuclear neutrophil
PRRs: Pattern recognition receptors
PS: Phosphatidyl serine
PYD: Pyrin domain
RAGE: Receptor for advanced-glycation end-products
RIP: Receptor-interacting protein kinase
ROS: Reactive oxygen species
Nec-1: Necrostatin-1
SODD: Silencer of death domains
TIM: T-cell immunoglobulin domain and mucin domain
TNFR: Tumor necrosis factor receptor
TRADD: TNF-α receptor-associated death domain
TRAF: TNF-α receptor associated factor
